# Implications of edge cases for antimicrobial susceptibility predictions for rifampin, delamanid, and pretomanid with the WHO *Mycobacterium tuberculosis* mutation catalog

**DOI:** 10.1128/spectrum.01821-26

**Published:** 2026-08-26

**Authors:** Noud Hermans, Jody Phelan, Rita Sorrentino, Fenja Boysen, Norelle L. Sherry, Paolo Miotto, Daniela Maria Cirillo, Leonid Chindelevitch, Timothy C. Rodwell, Ivan Barilar, Stefan Niemann, Sönke Andres, Alexander Mischnik, Kristy Horan, Katherine Bond, Maria Globan, Mor Rubinstein, Yelena Losev, Taane G. Clark, Satoshi Mitarai, Altyn Iskakova, Andrii Slyzkyi, Claudio U. Köser, Kristin Kremer, Richard Anthony

**Affiliations:** 1 Diagnostics Team, Division of Tuberculosis Elimination & Health Systems Innovations, KNCV Tuberculosis Foundation, The Hague, The Netherlands; 2 Department Bacterial Surveillance and Response, Center for Infectious Disease Control, Diagnostics and Laboratory Surveillance, National Institute for Public Health and the Environment (RIVM), Bilthoven, The Netherlands; 3 Department of Infection Biology, London School of Hygiene & Tropical Medicine, London, United Kingdom; 4 Emerging Bacterial Pathogens Unit, IRCCS San Raffaele Scientific Institute, Milan, Italy; 5 National and WHO-Supranational Reference Laboratory for Mycobacteria, Leibniz Lung Center, Borstel, Germany; 6 Microbiological Diagnostic Unit Public Health Laboratory, Department of Microbiology & Immunology, University of Melbourne at the Peter Doherty Institute for Infection & Immunity, Melbourne, Victoria, Australia; 7 Department of Infectious Diseases & Immunology, Austin Health, Heidelberg, Victoria, Australia; 8 Centre for Pathogen Genomics, University of Melbourne, Melbourne, Victoria, Australia; 9 MRC Center for Global Infectious Disease Analysis, School of Public Health, Imperial College London, London, United Kingdom; 10 Division of Pulmonary, Critical Care, Sleep Medicine, and Physiology, University of California San Diego, San Diego, California, USA; 11 Molecular and Experimental Mycobacteriology, Research Center Borstel, Borstel, Germany; 12 Partner site Hamburg-Lübeck-Borstel-Riems, German Center for Infection Research, Borstel, Germany; 13 Faculty of Medicine, Institute of Medical Microbiology and Hygiene, Medical Center, University of Freiburg, Freiburg, Germany; 14 Mycobacterium Reference Laboratory, Victorian Infectious Diseases Laboratory, at The Peter Doherty Institute for Infection & Immunity, Melbourne, Victoria, Australia; 15 Department of Microbiology, Royal Melbourne Hospital, Parkville, Victoria, Australia; 16 Department of Infectious Diseases, University of Melbourne at the Peter Doherty Institute for Infection & Immunity, Melbourne, Victoria, Australia; 17 National Public Health Laboratory, Public Health Directorate, Ministry of Health, Tel Aviv, Israel; 18 Faculty of Epidemiology and Population Health, London School of Hygiene and Tropical Medicine, Keppel Street, London, United Kingdom; 19 Department of Mycobacterium Reference and Research, Research Institute of Tuberculosis, Japan Anti-Tuberculosis Association, Kiyose, Tokyo, Japan; 20 Department of Basic Mycobacteriology, Nagasaki University Graduate School of Biomedical Sciences, Nagasaki, Japan; 21 KNCV Tuberculosis Foundation Kyrgyzstan, Bishkek, Kyrgyzstan; 22 National Center for Phthisiology, Bishkek, Kyrgyzstan; 23 Department of Genetics, Cambridge University, Cambridge, United Kingdom; ICON plc, London, United Kingdom

**Keywords:** *Mycobacterium tuberculosis*, whole-genome sequencing, WHO mutation catalog, rifampin, delamanid, pretomanid

## Abstract

The WHO mutation catalog is the reference for genotypic antimicrobial susceptibility predictions (ASPs) for *Mycobacterium tuberculosis*. To increase sensitivity, WHO endorsed additional grading rules to classify mutations with insufficient experimental evidence. For rifampin, this includes any non-silent *rpoB* mutation in the rifampin resistance-determining region (RRDR). For delamanid and pretomanid, the rules include loss-of-function (LoF) variants in six resistance genes, including *fbiC*. Here, we describe three edge cases in which these additional grading rules overcalled resistance. The first case was a low-frequency *rpoB* variant in RRDR caused by a sequencing artifact. The second case involved a tandem repeat region starting at the end of *fbiC*. Because the H37Rv reference genome carries 2.7 repeats, deletion of one or two full repeats caused mapping errors, making these deletions appear as minority variants that trigger the *fbiC* LoF rule. However, assembly showed that these deletions did not affect the *fbiC* coding region because the remaining repeat substituted for the deleted repeats. The third case was a *fbiC* frameshift in lineage 4.6.1 Uganda genotype strains, caused by a 19 bp duplication upstream of the same tandem repeat region. Although this frameshift affects the *fbiC* coding region and meets the *fbiC* LoF rule, sequence assembly revealed only a modest impact on the end of *fbiC*. Although catalog performance remains high, WHO and others have acknowledged that, in specific situations, exceptions to its additional grading rules may result in unintended overcalling of resistance. Thus, systematic external quality control and communication of edge cases are essential.

**IMPORTANCE:**

Antimicrobial susceptibility predictions (ASPs) for *Mycobacterium tuberculosis* using next-generation sequencing and the WHO mutation catalog are increasingly used for clinical decision-making. This avoids systematic false-resistant results of real-time PCR- or hybridization-based ASP assays caused by mutations that do not confer resistance (e.g., synonymous rifampin resistance-determining region [RRDR] mutations) and, potentially, subsequent inappropriate treatment adjustments. However, we demonstrate that false-resistant results occur even using the WHO mutation catalog for rifampin, delamanid, and pretomanid. Similar exceptions linked to the additional grading rules of the catalog are possible for other drugs, underlining the importance of monitoring the performance of the catalog continuously.

## OBSERVATION

The second edition of the WHO mutation catalog for *Mycobacterium tuberculosis* is the reference for interpreting mutations for genotypic antimicrobial susceptibility predictions (ASPs), including for rifampin, delamanid, and pretomanid (1–4). Broadly speaking, WHO used two approaches for classifying variants as markers of resistance (i.e., depending on the quality of evidence, associated with resistance [Group 1] or associated with resistance-interim [Group 2]). For some mutations, the available evidence was sufficient to grade them as Group 1 or 2. However, to boost the sensitivity for drugs with many individually rare resistance mutations, WHO endorsed additional grading rules according to which all mutations with certain characteristics were classed as Group 2 mutations—even if not previously observed (3, 5). The additional grading rule for rifampin applies to any non-silent *rpoB* mutation in codons 426–452, the rifampin resistance-determining region (RRDR). For delamanid and pretomanid, the additional grading rules cover any loss-of-function (LoF) variant (i.e., full-gene deletions, frameshifts, mutations abolishing the start codon, or introducing premature stop codons) in six resistance genes, including *fbiC* (3, 6, 7). In this study, we present three edge cases in which these additional grading rules overcalled resistance.

The first example concerns a T to A change at position 1346 of *rpoB*, resulting in a Leu449Gln change in RRDR. It was first observed at an approximate allele frequency of 5% by two participants of an external quality assessment (EQA) scheme for whole-genome sequencing (WGS) and reported as potentially emerging rifampin resistance because of the aforementioned additional grading rule (8). The presence of this variant in the Illumina sequencing reads was confirmed, and no evidence of sequencing bias or contamination was observed, but both isolates were susceptible by phenotypic antimicrobial susceptibility testing (AST) (Supplemental results S1 and Table S1) (8).

**Fig 1:**
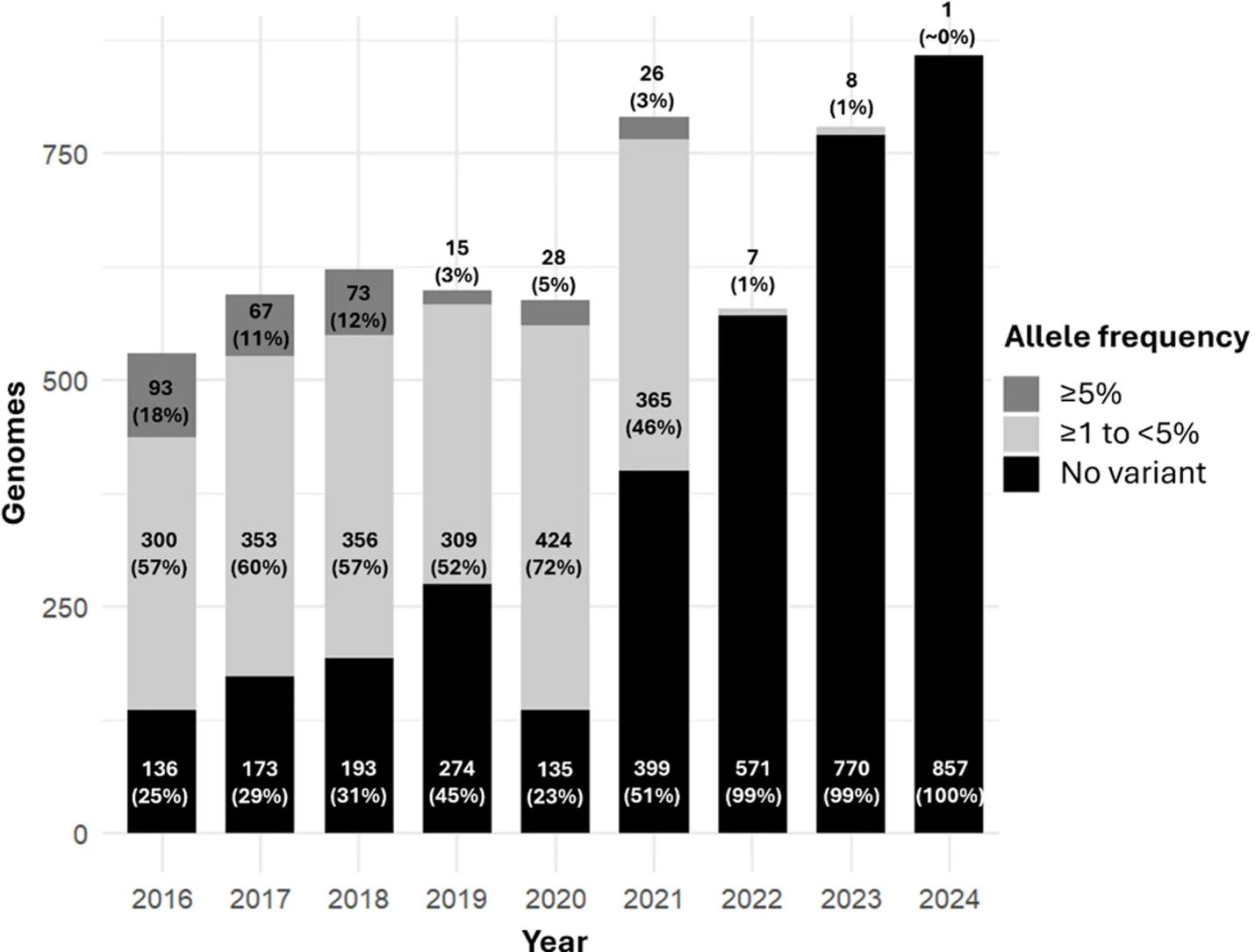
Overview of frequency of *rpoB* 1346 T to A change in the Dutch WGS reference database. Only genomes with a coverage ≥50× were included. A more detailed breakdown can be found in Table S2.

A review of the Dutch WGS reference database at the National Institute for Public Health and Environment (RIVM) for 2016–2021 revealed that 57% (95% CI 56%–59%) of the genomes had this nucleotide change at an allele frequency ≥1%, and 7% (95% CI 6%–8%) of genomes at an allele frequency ≥5% (Fig. 1; Table S2). By contrast, between 2022 and 2024, fewer than 1% (95% CI 0%–1%) of genomes showed this mutation at an allele frequency ≥1%. The observed nucleotide change was evenly distributed among all major *M. tuberculosis* complex lineages. AST results were available for 1,115 of 5,933 isolates with the nucleotide change (allele frequency ≥ 1%) during the study period. All were susceptible, except for 14 isolates with a resistance mutation elsewhere (Table S1). Even though AST is prone to misclassifying *rpoB* mutations conferring borderline MIC increases as susceptible, a resistance rate of 0% (95% CI 0%–0%) represented strong evidence that these isolates were truly susceptible (3, 6, 7).

Notably, the transition to in-house sequencing at RIVM just before 2022 suggests that the mutation represents a sequencing artifact related to the previous workflow (Supplemental methods and results, S1 and Table S2). Moreover, the instructions for use of the WHO-recommended GenoScreen Deeplex Myc-TB targeted next-generation sequencing (tNGS) assay consider this *rpoB* position as noisy, and the associated analysis pipeline raises the threshold for variant calling at this position to an allele frequency of 10% compared to as low as 3% elsewhere (9). Using the WGS analysis pipeline at RIVM, 0.6% (95% CI 0.5%–0.7%) of all genomes reviewed had the variant at ≥10%, with 19% being the highest observed allele frequency, highlighting the importance of systematically screening for noisy positions and establishing appropriate, context-dependent thresholds for the specific analysis pipeline used (Table S2).

The second edge case example relates to a tandem repeat region that begins at the end of *fbiC* and spans 2.7 repeats in the H37Rv genome (Fig. S3) (10). Soliman et al. reported four *Mycobacterium bovis* strains, each with a deletion of one of these repeat copies and interpreted this as molecular evidence of delamanid resistance (11). A separate study also identified the same deletion in four *M. tuberculosis* strains (12). We found that the number of repeats in this region is strongly correlated with *M. tuberculosis* complex lineage (Table 1). However, deletions of one or both full repeats do not affect the coding region of *fbiC* because the remaining full or partial repeat substitutes for the deleted nucleotides. Yet, because variant callers typically position deletions at the leftmost repeat, these deletions are annotated by SNPeff (13) as frameshifts, thereby triggering the LoF additional grading rule and overcalling resistance to delamanid and pretomanid. In other pipelines, including the one used at RIVM, single-copy deletions appear as minority variants (~25%) due to mapping bias and are consequently not called. However, they can still be inferred using SNPs downstream of *fbiC* (Fig. S3). Using these SNPs, we estimated the frequency of the single-copy and double-copy deletions to be 27% and 0.2%, respectively, in the Dutch WGS reference database (Table 1).

**TABLE 1:** Frequency of *fbiC* repeat deletions in the Dutch WGS reference database^*a*^

MTBC genotype	Total	1-Copy deletion^*b*^	2-Copy deletion	Wild type
Lineage 1	731	37 (5%)	0 (0%)	694 (95%)
Lineage 2	848	31 (4%)	2 (0.2%)	815 (96%)
Lineage 3	1,054	1,053 (100%)	1 (0%)	0 (0%)
Lineage 4	4,286	515 (12%)	9 (0.2%)	3,762 (88%)
Lineage 5	39	0 (0%)	0 (0%)	39 (100%)
Lineage 6	57	14 (27%)	0 (0%)	43 (76%)
Lineage 7	7	7 (100%)	0 (0%)	0 (0%)
*M. bovis*/BCG	305	305 (100%)	0 (0%)	0 (0%)
Total	7,329	1,962 (27%)	12 (0.2%)	5,355 (73%)

^*a*^ For the 2015–2025 period. See Fig. S3 for more details.

^*b*^ A mixed SNP at position 1,305,593 was used to infer the presence of the 1-copy deletion (more details in Supplemental  S1).

The final edge case is a *fbiC* frameshift in lineage 4.6.1 Uganda genotype strains caused by a 19 bp duplication upstream of the tandem repeat region just discussed (Fig. S4). Unlike the above repeat deletions, this frameshift has a real effect on the *fbi*C coding region, and the WHO LoF rule applies. However, assembling the corresponding sequence revealed only a modest impact on the end of *fbiC* (Fig. S4), which likely explains why frameshift mutants tested susceptible to delamanid and pretomanid in four laboratories (Table S1).

ASPs using the WHO mutation catalog demonstrated a good specificity for all drugs and excellent sensitivity for predicting pan-susceptibility to first-line drugs, resulting in a drastic reduction in the use of AST in several well-resourced countries with low prevalences of drug-resistant tuberculosis (1, 2, 4, 5, 14–17). Importantly, using the catalog for interpreting tNGS and WGS data avoids systematic false-resistant results of real-time PCR- or hybridization-based ASP assays caused by mutations that do not confer resistance (i.e., Group 4 or 5 mutations in the catalog, such as synonymous mutations in RRDR for rifampin, or *gyrA* Ala90Gly for levofloxacin and moxifloxacin) (3, 18–20). Nevertheless, WHO and others have acknowledged that, in specific situations, exceptions to its additional grading rules may result in unintended overcalling of resistance—particularly for rifampin, for which WHO endorsed a composite reference standard of ASP and AST to avoid the misclassification of borderline *rpoB* mutants (3, 6, 7, 21–24). Indeed, based on our aforementioned EQA results and information from GenoScreen, the second edition of the mutation catalog featured a warning about the *rpoB* 1346 T to A variant (3, 8, 9). Our study confirms that this mutation is likely a sequencing workflow artifact. A warning about the bioinformatic artifact linked to the *fbiC* tandem repeat will be included in the upcoming third edition of the WHO mutation catalog. The neutral *fbiC* duplication, the first true exception to an additional grading rule with strong experimental support, will have to be considered in the subsequent edition because the relevant AST results were not available in time for the third edition to include them. Version 6.6.6 of TB-Profiler incorporated updates to address the first two edge cases (Fig. S5). The performance of the catalog should be monitored during future EQA rounds and routine use to identify and rapidly communicate edge cases.

## Data Availability

Whole-genome sequencing data are available in the Sequence Read Archive of the National Center for Biotechnology Information and the European Nucleotide Archive as FASTQ files. Sample-specific accession numbers and metadata can be found in Table S1.

## SUPPLEMENTAL MATERIAL

Supplemental methods and results spectrum.01821-26-s0001.pdf Supplemental methods and results.

Table S1 spectrum.01821-26-s0002.xlsx Details on AST per isolate.

ASM does not own the copyrights to Supplemental Material that may be linked to, or accessed through, an article. The authors have granted ASM a non-exclusive, world-wide license to publish the Supplemental Material files. Please contact the corresponding author directly for reuse.
