# Supplementary material for: Implications of edge cases for antimicrobial susceptibility predictions for rifampin, delamanid, and pretomanid with the WHO Mycobacterium tuberculosis mutation catalog
Source: Microbiol Spectr. 2026 Aug 26;14(10):e01821-26. doi: 10.1128/spectrum.01821-26 (PMC13689116; doi:10.1128/spectrum.01821-26)
Supplement: Supplementary file 1 [file spectrum.01821-26-s0001.pdf]

Supplementary Methods

Downstream bioinformatic analyses of low frequency *rpoB* variant

Sequencing setup used from 2016 to 2024

In the period 2016- 2021 sequencing of all *Mycobacterium tuberculosis* isolates at the National Institute for Public Health and Environment (RIVM) was outsourced. During this period multiple sequencing setups (device + chemistry) were utilized:

- **2016–2019:** The HiSeq 2000 instrument (Illumina, San Diego, CA, United states) was used in combination with the Illumina XT library prep kit (Illumina).
- **2020–2021:** The NovaSeq 6000 instrument (Illumina) was used in combination with the Illumina XT library (Illumina).

By the start of 2022, the RIVM had transitioned to in-house sequencing, the library preparation method and the applied sequencing devices used were as follows:

- **2022–2023:** The NextSeq 500/550 instrument (Illumina) was used in combination with the Illumina DNA prep (Illumina).
- **2024:** The NextSeq 2000 instrument (Illumina) was used in combination with the Illumina DNA prep kit (Illumina).

NCBI BLAST analysis of reads containing the *low frequency rpoB variant*

First, the reads were mapped to the *M. tuberculosis* H37Rv reference genome (accession AL123456.3) using CLC Genomics Workbench (version 25.0.1) (Qiagen, Hilden, Germany). Reads containing the position 1346 codon 449, Leu > Gln *rpoB* mutation were extracted and analyzed using the BLAST nucleotide database BLAST: Basic Local Alignment Search Tool (1). During the BLAST search, MTBC species were excluded from the search.

Search query BLAST (low frequency variant nucleotide in bold):

TCCGGGCGGCGTCGAGGTGCCGGTGGAAACCGACGACATCGACCACTTCGGCAACCGCCGCCTGCGTACGGTCGG
CGAGCTGATCCAAAACCAGATCCGGGTCGGCATGTCGCGGATGGAGCGGGTCGCCGCGATCAAGGAGTTCTTCGGC
ACCAGCCAGCTGAGCCAATTCATGGACCAGAACAACCCGCTGTCGGGGTTGACCCACAAGCGCCGAC**A**GTCGGCG
CTGGGGCCCGGCGGTCTGTCACGTGA

Assessment for potential sequencing bias

To investigate the effect of quality filtering on the presence of the *rpoB* low-frequency variant, we assessed the data for potential sequencing bias. RStudio (version 4.4.2) was used to randomly sample ten WGS datasets from the 3,844 isolates that contained the low-frequency variant in the *rpoB* gene (~5%, *rpoB* position 1346, codon 449 (RRDR), T > A, Leu > Gln) in the Dutch WGS reference database at the RIVM.

First, the reads were mapped to the *M. tuberculosis* H37Rv reference genome (accession AL123456.3) using the Burrows–Wheeler Aligner (BWA, version 0.7.19) (2). Subsequently, the output SAM files were converted to and sorted in BAM format using Samtools (version 1.22.1) (3), which were used for further analysis.

To extract base counts for the positive and negative strands, base and read quality scores, and base in-read position data, Pysam (version 0.23.3) (4) was used. All output data were captured in a .csv file and analyzed and visualized using RStudio. Within RStudio, the ggplot2 (version 3.5.2) (5) and reshape2 (version 1.4.4) (6) packages were used.

Genomic visualization of the *fbiC* coding and repeat regions

Detailed analysis of isolates with the single repeat deletion was conducted using CLC Genomics Workbench (version 25.0.1) (Qiagen) and TRF (v4.09.1) (7). In CLC Genomics Workbench, reads were mapped to the *M. tuberculosis* H37Rv reference genome (accession AL123456.3) using the built-in "map reads to reference" tool to assess coverage and variant characteristics.

Subsequently, TRF was used to analyze a 300 bp region starting 100 bp upstream and ending 200 bp downstream of the *fbiC* coding region. CLC Genomics Workbench was then used to visualize the results. The built-in "create alignment" tool was used to align all repeats identified within the 300 bp region to verify sequence identity.

Antimicrobial susceptibility testing (AST) rifampin, delamanid, and pretomanid at RIVM

AST for **rifampin** (BD Bactec MGIT 960 SIRE kit) (Beckton Dickenson (BD), Franklin Lakes, NJ, USA) was performed by Mycobacterial growth indicator tubes (MGIT) breakpoint testing. MGIT tubes (BD) were prepared according to the manufacturer’s protocols by adding 800 µl of OADC Middlebrook enrichment (BD), followed by adding rifampin. Before 2022, samples were tested at the critical concentration of 1.0 µg/ml, whereas after this period the newly WHO-recommended critical concentration of 0.5 µg/ml was used (8).

The Sensititre UKMYC5 plates (Thermo Scientific, Waltham, MA, USA) were used to perform AST for **delamanid** as previously described and interpreted using 0.12 µg/ml as the critical concentration (9, 10). AST for **pretomanid** was done using MGIT breakpoint testing. From a stock solution of 10,000 µg/ml of pretomanid (Sigma-Aldrich, St Louis, MO, USA) in DMSO (Thermo Scientific), a working dilution was prepared by diluting the stock solution in water to a concentration of 200 µg/ml. The MGIT tubes were prepared according to the manufacturer's protocols by adding 800 µl of OADC Middlebrook enrichment, followed by pretomanid (working dilution) to a final concentration of 0.5 and 2.0 µg/ml in the MGIT tube, as recommended by the WHO (11). Subsequently, 500 µl of a MTBC-positive MIGT culture was inoculated in the pretomanid-containing MGIT tube. All laboratories performed MGIT testing for pretomanid at both 0.5 and 2 µg/mL, except for the RIVM, where testing was performed only at 0.5 µg/mL.

AST at the remaining sites

The EUCAST reference method, MGIT, and/or Sensititre UKMYC5/UKMYC6 plates were used as previously described (9,12).

Supplementary Results

Effect of sequencing instrument on low frequency variant prevalence

Because sequencing data generated before the transition to in-house sequencing at RIVM were produced on two different Illumina platforms, we assessed whether low-frequency variant detection was affected by sequencing platform. The HiSeq 2000 and NovaSeq 6000 use different data acquisition methods, with HiSeq 2000 using four-channels and NovaSeq 6000 using two-channels. The average annual number of detected low-frequency variants was broadly comparable between platforms. For variants detected at ≥1% to <5% allele frequency, the annual averages were 330 for HiSeq 2000 and 395 for NovaSeq 6000. For variants detected at ≥5% allele frequency, the corresponding averages were 62 and 27, respectively.

NCBI BLAST analysis of reads containing the *low-frequency rpoB variant*

No 100% matches were found in species not belonging to the MTBC. Thus, if the low-frequency variant is caused by contamination, it likely originates from a currently undescribed non-MTBC species.

Assessment of *rpoB* minority variant reads for strand bias

A comparative analysis of the distribution of the reference and alternative alleles (low-frequency *rpoB* variant) at *rpoB* position 1346, codon 449, across the positive and negative DNA strands was performed. As illustrated in Figure S1, strand bias was not observed, as both the reference and alternative alleles were detected on both positive and negative strands.

Furthermore, no significant difference in the proportional distribution of these alleles between the positive and negative strands was observed across the randomly selected samples (chi-squared test, $p = 0.893$).

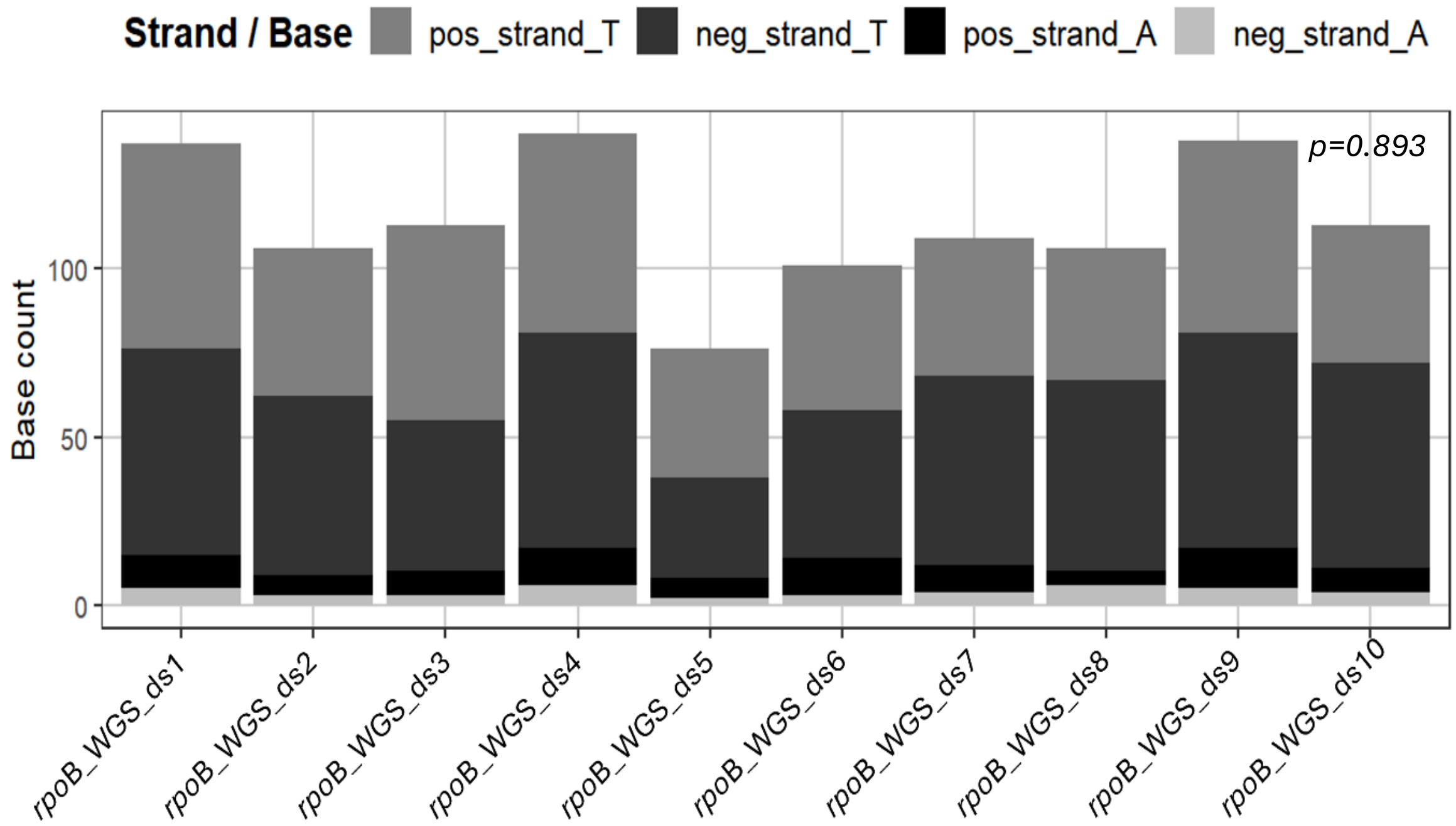

Figure S1: Distribution of strand-specific (pos_strand and neg_strand) base counts for the reference allele (T) and alternative allele (A) at rpoB position 1346 (codon 449) across ten randomly selected WGS MTBC datasets (rpoB_WGS_ds1 to rpoB_WGS_ds10). Ten samples were included in the analysis (≥50× coverage at rpoB position 1346). A chi-squared test was used to compare the proportions of the reference and alternative alleles between the positive and negative strands.

Assessment of *rpoB* minority variant reads for base specific and read quality

As illustrated in Figure S2A, a significant difference in base-specific quality was observed between the reference and alternative alleles (Welch's t-test, p = 0.0005). The alternative allele (low-frequency *rpoB* variant) exhibited a higher mean base Q score than the reference allele.

Conversely, no significant difference in read quality was observed between reads containing the reference base and those containing the alternative base (Figure S2B, Welch's t-test, p = 0.96).

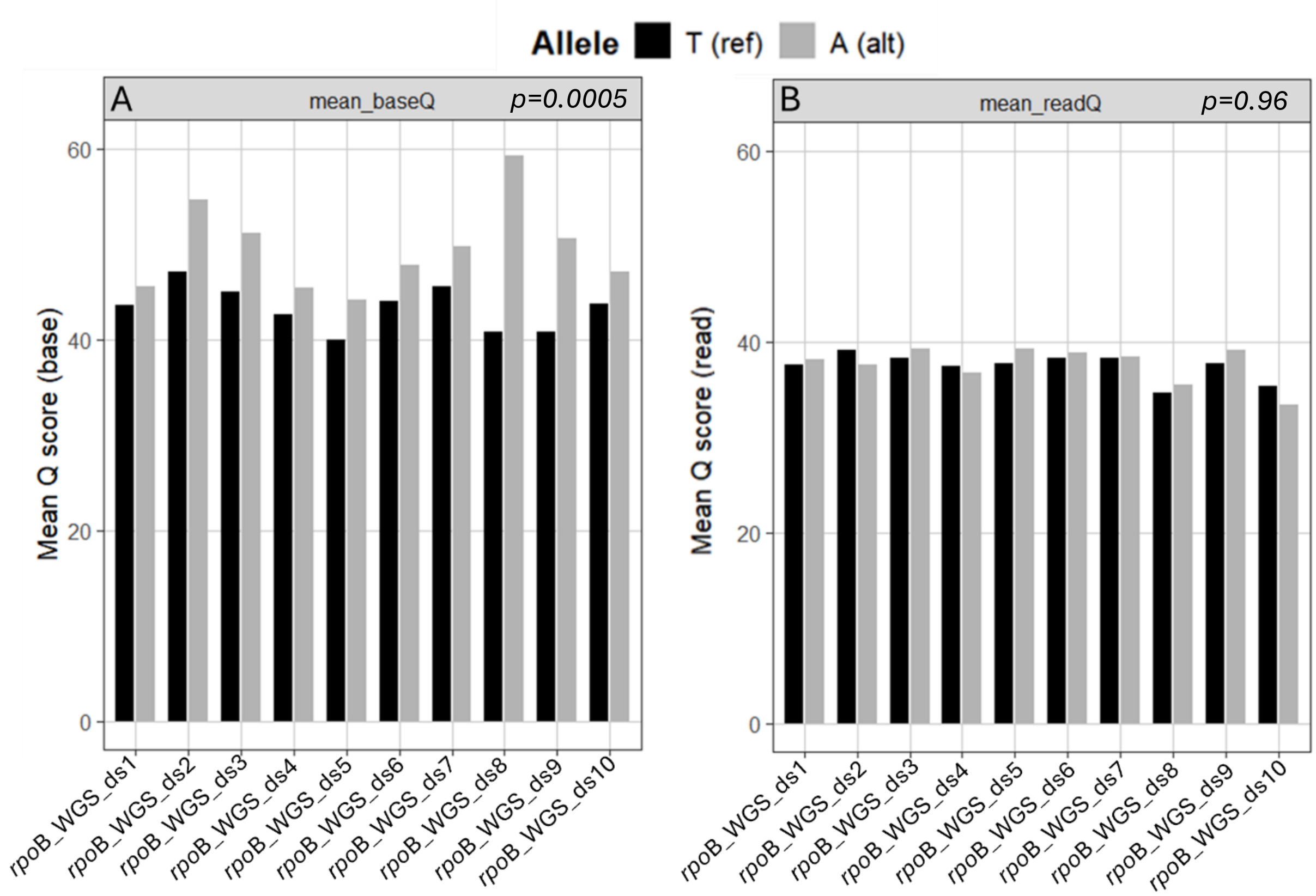

*Figure S2**: (A) Mean base quality** for the reference allele (T) and alternative allele (A) at rpoB position 1346 (codon 449) in the M. tuberculosis H37Rv reference genome across ten randomly selected WGS MTBC datasets (rpoB_WGS_ds1 to rpoB_WGS_ds10). **(B) Mean read quality** for reads containing the reference allele (T) and the alternative allele (A) at rpoB position 1346 (codon 449) in the M. tuberculosis H37Rv reference genome across ten randomly selected WGS MTBC datasets (rpoB_WGS_ds1 to rpoB_WGS_ds10). Ten samples were included in the analysis (≥50× coverage at rpoB position 1346).*

Assessment of *rpoB* minority variant reads for position bias

The occurrence of low-frequency variants at the start (first 25 bp), middle (variable), and end (last 25 bp) of the sequencing reads were examined to assess position bias. Data from all ten datasets were pooled, and the frequency read per segment was calculated.

The alternative allele (low-frequency variant) was observed across all three segments and was not disproportionately represented at either the start or the end of the reads. In total, the low-frequency variant was observed 123 times across the ten datasets: 26 occurrences in the first 25 bp (21%) and 15 occurrences in the last 25 bp (12%). Most observations were in the middle of the read, with 82 occurrences (67%).

Antimicrobial susceptibility testing for rifampin of isolates with a low-frequency *rpoB* mutation

As discussed in the main text, 1,101 of the 1,115 isolates containing the low-frequency variant were phenotypically classified as susceptible to rifampin. All 14 resistant isolates harbored an additional high-confidence mutation in the *rpoB* RRDR region (Table S1).

Prevalence of low-frequency *rpoB variant* in the Dutch WGS reference database

Table S2: Overview of the frequency of the rpoB 1346 T > A variant in the Dutch WGS reference database.

Year	2016	2017	2018	2019	2020	2021	2022	2023	2024	2016–2021	2022–2024	2016–2024
Total	529 (8.9%)	593 (10.0%)	622 (10.5%)	598 (10.1%)	587 (9.9%)	790 (13.3%)	578 (9.7%)	778 (13.1%)	858 (14.5%)	3,719 (62.7%)	2,214 (37.3%)	5,933 (100.0%)
No variant	136 (25.7%)	173 (29.2%)	193 (31.0%)	274 (45.8%)	135 (23.0%)	399 (50.5%)	571 (98.8%)	770 (99.0%)	857 (99.9%)	1,310 (35.2%)	2,198 (99.3%)	3,508 (59.4%)
1% ≤ AF < 5%	300 (56.7%)	353 (59.5%)	356 (57.2%)	309 (51.7%)	424 (72.2%)	365 (46.2%)	7 (1.2%)	8 (1.0%)	1 (0.1%)	2,107 (56.7%)	16 (0.7%)	2,123 (35.8%)
5% ≤ AF < 10%	80 (15.1%)	58 (9.8%)	67 (10.8%)	13 (2.2%)	26 (4.4%)	25 (3.2%)	0 (0.0%)	0 (0.0%)	0 (0.0%)	249 (6.9%)	0 (0.0%)	249 (4.2%)
10%≤ AF < 20%	13 (2.5%)	9 (1.5%)	6 (1,0%)	2 (0.3%)	2 (0.4%)	1 (0.1%)	0 (0.0%)	0 (0.0%)	0 (0.0%)	33 (1.1%)	0 (0.0%)	33 (0.6%)
highest AF (%)	19.4	16.7	18.9	18.0	13.2	12.2	1.7	3.1	1.1	19.4	3.1	19.4

Genomic visualization of the *fbiC* coding and repeat region

Detailed analysis of isolates with repeat deletions was performed using CLC Genomics Workbench (version 25.0.1) and TRF (v4.09.1). In the *M. tuberculosis* H37Rv reference genome (GenBank accession AL123456.3), the region near the 3′ end of the *fbiC* gene contains two full repeats followed by one imperfect partial repeat, all of which contain a stop codon (Figure S4A). The repeat region starts 7 bp upstream of the end of the *fbiC* gene.

We observed three options:

1. **Reference sequence with two full repeats and one partial repeat (Figure S3A).**
2. **One full copy deletion (Figure S3C).**
3. **Double full copy deletion (Figure S3D).**

In variants with a single repeat deletion, the pipeline detected an apparent frameshift at ~25% allele frequency. This ~25% allele frequency was a mapping artefact: only reads spanning the deletion supported the variant, while reads starting within or downstream of the repeat aligned normally, resulting in the deletion being called at low frequency. In double repeat deletions, the frameshift was observed at 100% frequency, as sequencing reads were of sufficient length to cover the entire affected region. However, in both variants, the remaining repeat substituted the deleted repeat(s), and the *fbiC* coding region remained unchanged (Figure S3A, C, and D).

All variants with repeat deletions also showed a SNP downstream of the *fbiC* gene. This SNP appeared at position 1,305,593 in single repeat deletions and at position 1,305,531 in double repeat deletions. However, these were not true SNPs but mapping artefacts.

Comparison of the full and partial repeat sequences revealed a single nucleotide difference ((partial) repeat position 37, C > G) (Figure S3B). When sequencing data from isolates missing one or two full repeats were mapped to the reference genome, the partial repeat aligned to a different position. This shift caused the nucleotide difference to appear as a SNP at different genomic coordinates (Figure S3A, C, and D, green arrows).

These SNPs are not associated with drug resistance but instead serve as reliable indicators for the presence of single and double repeat deletions, as the deletions themselves are not consistently detected by the pipeline.

In summary, three variants were observed: the reference structure, a single repeat deletion, and a double repeat deletion. In all cases, the *fbiC* coding sequence remained unchanged.

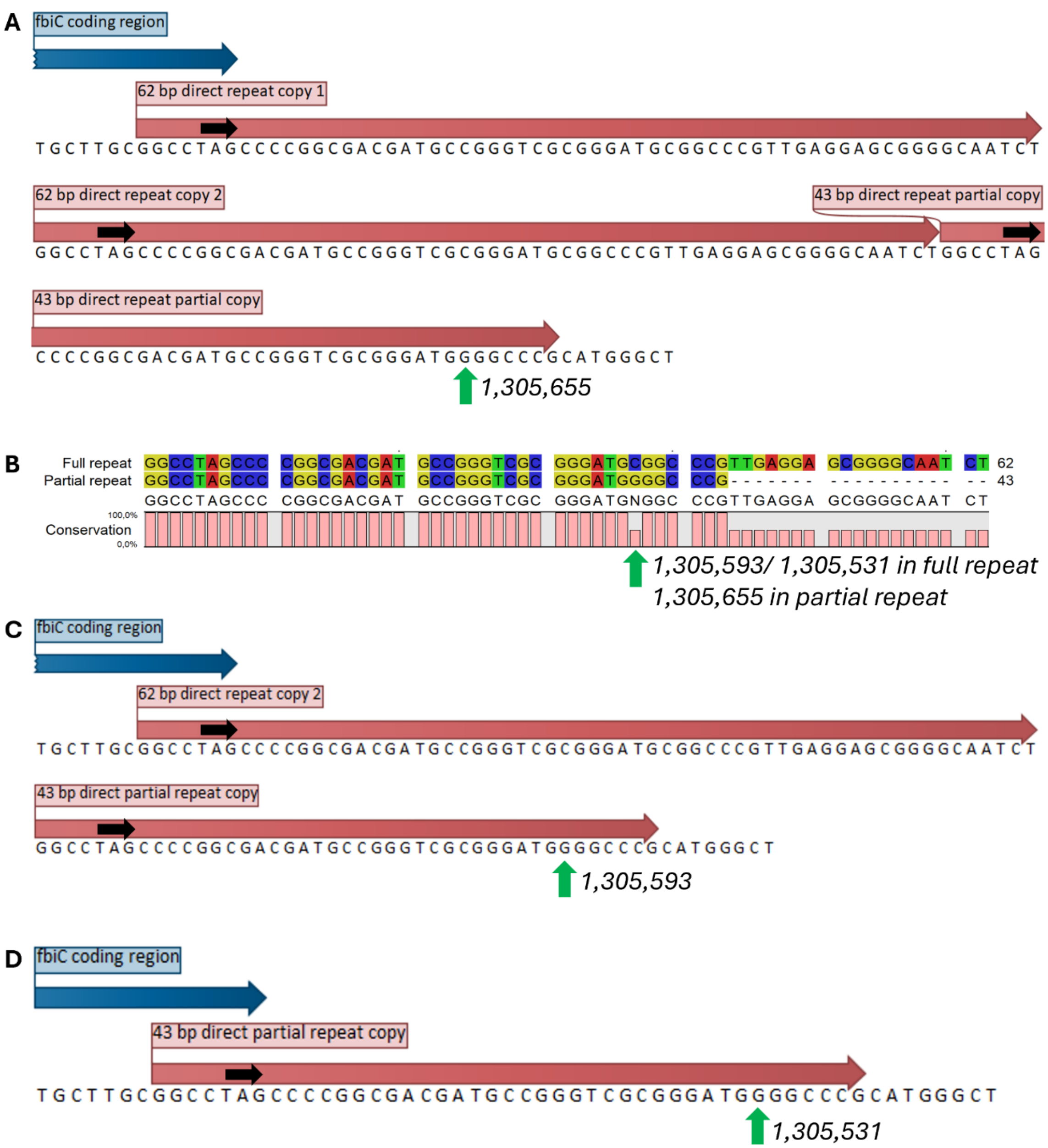

Figure S3: Visual representation of the 3′ end of the fbiC coding region (blue arrow) and the corresponding repeat series (red arrows) in the M. tuberculosis H37Rv reference genome.
(A) *A series of 2.7 repeats (red arrows) starts 7 bp upstream of the end of the fbiC gene (blue arrow). All repeat copies contain a stop codon (black arrow).*
(B) *Comparison of the full and partial repeats shows a single nucleotide difference (green arrow; position 1,305,531, corresponding to position 37 within the repeat; C > G).*

(C) *Strains with a single full repeat deletion. The fbiC coding region (blue arrow) remains unchanged, and the lost stop codon is substituted. Additionally, an artificial "mixed SNP" is observed at position 1,305,593 (green arrow).*

(D) *Strains with a double full repeat deletion. The fbiC coding region (blue arrow) remains unchanged, and the lost stop codon is substituted. Additionally, an artificial SNP is observed at position 1,305,531 (green arrow).*

Search strategy for 62 bp (single) and 124 bp (double) repeat deletions

Screening of the Dutch database for isolates carrying the 62 bp repeat deletion showed that direct detection of the deletion was not feasible. As described above, loss of a single repeat leads to mapping artefacts, causing the deletion to appear at variable allele frequencies of approximately 25%. Because of this low allele frequency, the deletion was not detected by our routine analysis pipeline.

Isolates matching the reference genome contain two full copies of the repeat and one partial copy. In these isolates, no mapping artefacts or SNPs were observed in the associated region.

All isolates carrying the single 62 bp repeat deletion also showed a SNP at position 1,305,593 with a higher allele frequency (~75%). This SNP therefore served as a more reliable marker for isolates missing a single repeat. Our search strategy was therefore based on the presence of this SNP rather than the deletion itself.

To verify the accuracy of this approach, a subset of 25 samples previously classified as delamanid/pretomanid resistant due to the single repeat deletion was analyzed. All isolates in this subset carried the SNP, confirming the reliability of the strategy.

For isolates carrying the 124 bp double repeat deletion, the search strategy directly targeted the deletion, as this mutation was consistently detected at approximately 100% allele frequency. The results of this analysis are presented in the main text.

Insertion in the *fbiC* gene in Uganda sub-lineage strains

During screening of the Dutch database, we found five isolates with a 19 bp duplication in the *fbiC* gene at position 1,305,476. These isolates all belonged to the Uganda sub lineage. This duplication causes a frameshift that results in a modest extension of *fbiC* coding region (Figure S4). The presence of the duplication was confirmed by *de novo* assembly from long-read sequencing data (**ERS30300557**). To predict the effect of the insertion on nitroimidazole resistance 12 Uganda sub-lineage strains (Table S1) were subjected to AST for delamanid and pretomanid. All Uganda strains tested sensitive for both pretomanid and delamanid.

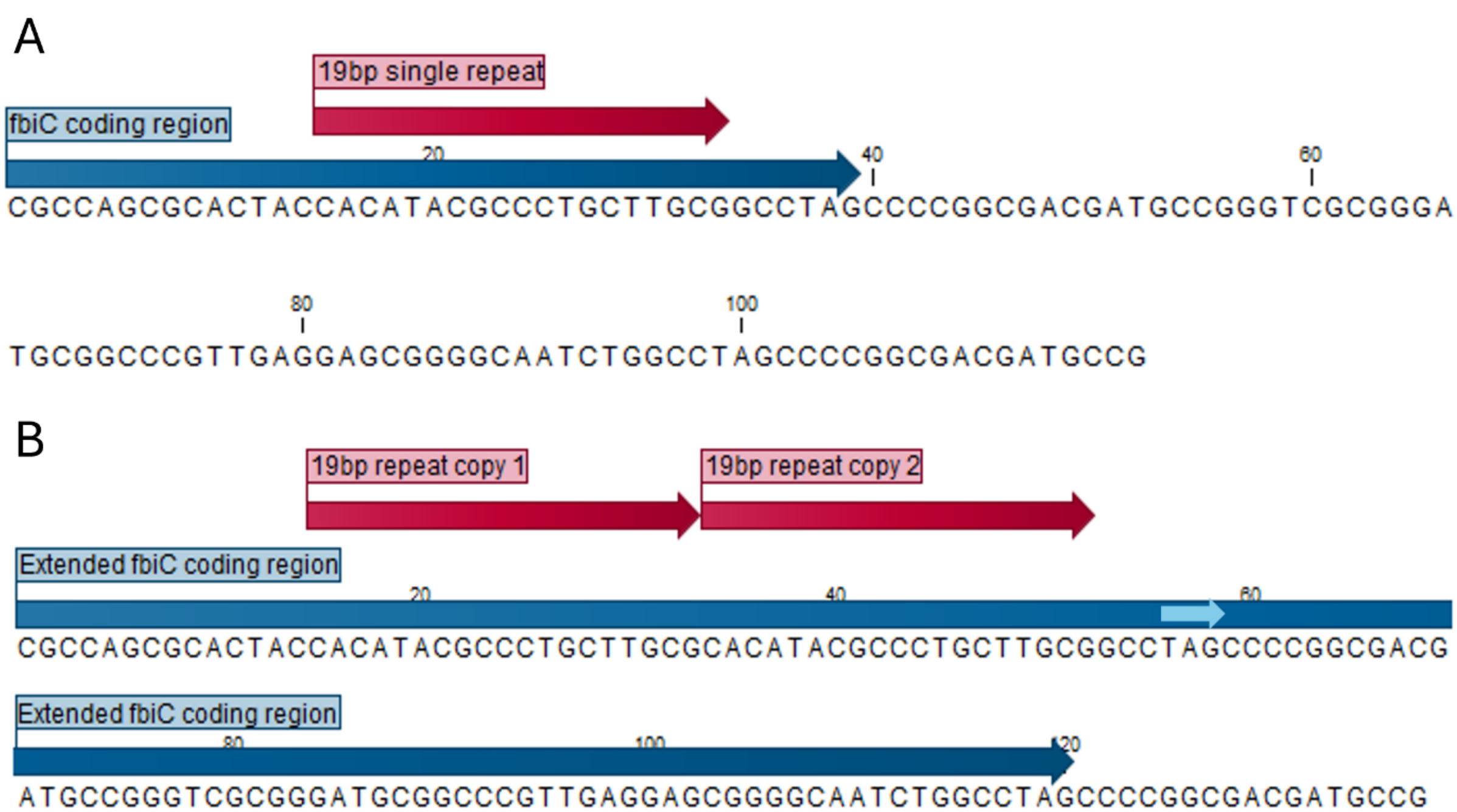

Figure S4: Visual representation of the 3' end of the fbiC coding region (blue arrow) and the 19 bp duplicated fragment (red arrows).
(A) *The M. tuberculosis H37Rv reference genome contains a single copy of the 19 bp fragment.*
(B) *Isolates belonging to the Uganda sub-lineage contain two copies (19 bp duplication), resulting in a frameshift.*

Updates to TB-Profiler to address the three edge cases

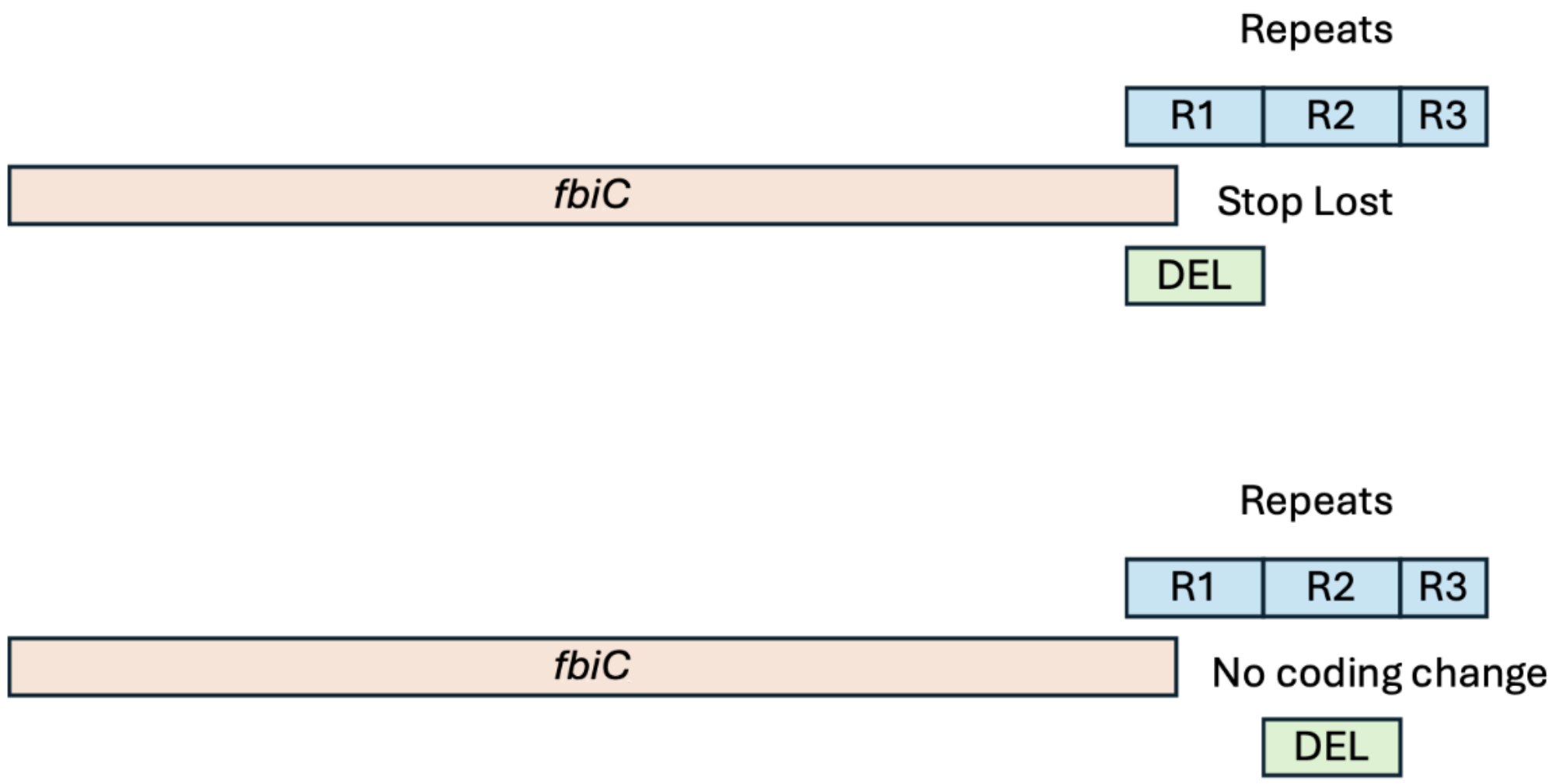

Figure S5: Schematic representation of the approach in TBProfiler to mitigate the fbiC bioinformatics artefact.

In the case of a tandem repeat deletion, TBProfiler addresses this issue by relocating the deletion outside the coding region (Figure S5). TBProfiler employs Delly (version 1.2.6) (13) for calling deletions. By default, this program positions the deletion at the leftmost repeat, which, in this instance, is within the coding region. SNPeff subsequently annotates this with a "stop_lost", resulting in interpreting it as resistant. In the current version of TBProfiler (v 6.6.6) (14) the deletion is positioned at the rightmost repeat, thereby moving it outside the coding region, calling the isolate susceptible. This process ensures that when a deletion is identified and it is not a tandem repeat deletion, it cannot be placed elsewhere and will be correctly annotated (13, 14).

References

1. Johnson M, Zaretskaya I, Raytselis Y, Merezhuk Y, McGinnis S, Madden TL. NCBI BLAST: a better web interface. Nucleic Acids Res. 2008;36 Suppl 2:W5-W9. doi:10.1093/nar/gkn201.

2. Li H. Aligning sequence reads, clone sequences and assembly contigs with BWA-MEM. arXiv. 2013 Mar 16;1303.3997.

3. Li H, Handsaker B, Wysoker A, Fennell T, Ruan J, Homer N, et al. The sequence alignment/map format and SAMtools. Bioinformatics. 2009;25(16):2078-2079. doi:10.1093/bioinformatics/btp352.

4. Bonfield JK, Marshall J, Danecek P, Li H, Ohan V, Whitwham A, et al. HTSlib: C library for reading/writing high-throughput sequencing data. Gigascience. 2021;10(2):giab007. doi:10.1093/gigascience/giab007.

5. Wickham H. ggplot2: elegant graphics for data analysis. Cham: Springer International Publishing; 2016. doi:10.1007/978-3-319-24277-4.

6. Wickham H. Reshaping data with the reshape package. J Stat Softw. 2007;21(12):1-20. doi:10.18637/jss.v021.i12.

7. Benson G. Tandem repeats finder: a program to analyze DNA sequences. Nucleic Acids Res. 1999;27(2):573-580. doi:10.1093/nar/27.2.573.

8. World Health Organization. Technical report on critical concentrations for drug susceptibility testing of isoniazid and the rifamycins: rifampicin, rifabutin and rifapentine. Geneva: World Health Organization; 2021. Licence: CC BY-NC-SA 3.0 IGO.

9. Rancoita PMV, Cugnata F, Gibertoni Cruz AL, Borroni E, Hoosdally SJ, Walker TM, et al. Validating a 14-drug microtiter plate containing bedaquiline and delamanid for large-scale research susceptibility testing of *Mycobacterium tuberculosis*. Antimicrob Agents Chemother. 2018;62(9):e00344-18. doi:10.1128/AAC.00344-18.

10. Epidemiological cut-off values for a 96-well broth microdilution plate for high-throughput research antibiotic susceptibility testing of *Mycobacterium tuberculosis*. Eur Respir J. 2022;60(4):2200239. doi:10.1183/13993003.00239-2022.

11. World Health Organization. Web Annex B. Critical concentrations for pretomanid and cycloserine: WHO policy statement. In: WHO operational handbook on tuberculosis. Module 3: diagnosis - rapid diagnostics for tuberculosis detection. 3rd ed. Geneva: World Health Organization; 2024. Licence: CC BY-NC-SA 3.0 IGO.

12. EUCAST AntiMycobacterial Susceptibility Testing Subcommittee. EUCAST reference protocol for MIC determination of anti-tuberculous agents against isolates of the *Mycobacterium tuberculosis* complex. Version 8.2. EUCAST; 2025 Jan 29.

13. Rausch T, Zichner T, Schlattl A, Stütz AM, Benes V, Korbel JO. DELLY: structural variant discovery by integrated paired-end and split-read analysis. Bioinformatics. 2012;28(18):i333-i339. doi:10.1093/bioinformatics/bts378.

14. Phelan JE, O'Sullivan DM, Machado D, Ramos J, Oppong YEA, Campino S, et al. Integrating informatics tools and portable sequencing technology for rapid detection of resistance to anti-tuberculous drugs. Genome Med. 2019;11(1):41. doi:10.1186/s13073-019-0650-x.
